# Ultrasonic Activation of Au Nanoclusters/TiO_2_: Tuning Hydroxyl Radical Production Through Frequency and Nanocluster Size

**DOI:** 10.3390/molecules30030541

**Published:** 2025-01-24

**Authors:** Takaaki Tsurunishi, Yuzuki Furui, Hideya Kawasaki

**Affiliations:** Department of Chemistry and Materials Engineering, Kansai University, 3-3-35, Yamate-cho, Suita 564-8680, Osaka, Japan; taka0902s3a_mz@outlook.jp (T.T.); k345862@kansai-u.ac.jp (Y.F.)

**Keywords:** sonocatalyst, TiO_2_, Au nanoclusters, reactive oxygen species, ultrasonic cavitation

## Abstract

This study explores the sonocatalytic activity of gold nanoclusters (Au NCs) combined with titanium dioxide (TiO_2_) nanoparticles, forming Au NCs/TiO_2_ composites. The hybrid material significantly enhances hydroxyl radical (•OH) generation under ultrasonic conditions, attributed to high-energy cavitation bubbles formed during ultrasonication. The effects of frequency (200, 430, and 950 kHz) and power were systematically evaluated on Au_144_/TiO_2_ composites, identifying 430 kHz as optimal for •OH production due to its efficient cavitation energy. Au_144_ NCs function as electron traps, reducing electron–hole recombination in ultrasonically activated TiO_2_, thereby improving charge separation and enhancing •OH generation. Size-dependent effects were also studied, showing an efficiency trend of Au_144_ > Au_25_ > plasmonic Au nanoparticles > bare TiO_2_. These findings highlight the importance of ultrasonication frequency and Au NC size in optimizing sonocatalytic performance in the Au NCs/TiO_2_ composites, providing valuable insights for designing advanced sonocatalysts with applications in chemical synthesis, environmental remediation, and biomedical fields.

## 1. Introduction

The hydroxyl radical (•OH) is an exceptionally potent oxidizing agent found in various environments, including natural waters, the atmosphere, and interstellar space. As one of the most important reactive oxygen species (ROS), •OH plays a pivotal role in numerous chemical processes, particularly in environmental chemistry and biochemistry. In environmental applications, advanced oxidation processes (AOPs) are widely employed in wastewater treatment to degrade pollutants effectively [1,2]. In the medical field, •OH radicals are utilized in innovative therapeutic approaches such as photodynamic therapy and chemodynamic therapy, which target cancer cells through oxidative damage within the tumor microenvironment [3,4]. Advancing methods for enhanced •OH radical generation is critical for expanding its potential applications in both environmental remediation and healthcare.

Photocatalytic materials absorb light with energy exceeding their band gap, generating excited electrons and holes. These charge carriers migrate to the surface of the material, where they act as reducing and oxidizing agents, facilitating redox reactions. For example, the reduction of oxygen or the oxidation of water results in the formation of reactive oxygen species (ROS), such as superoxide radicals and •OH. These ROS play a key role in degrading organic compounds. However, a significant portion of the excited electrons and holes recombine before contributing to the reaction, releasing energy as heat and light instead [5,6].

In addition to photocatalysis, sonocatalysis involves the activation of materials, known as sonocatalysts, by ultrasound waves in liquids through high-energy acoustic cavitation. The collapse of cavitation bubbles generates extreme heat and pressure, causing the pyrolytic cleavage of water molecules. This process thermally dissociates water into •OH and hydrogen atoms, often accompanied by sonoluminescence. The effectiveness of sonocatalysts arises from the inherent sonocatalytic activity of semiconductors [6,7]. In the medical field, sonocatalysts are pivotal in sonodynamic therapy, where ultrasound activates the sonocatalysts to produce ROS, selectively destroying tumor cells. Thus, sonocatalysts play a vital role in tackling environmental challenges and advancing medical therapies [8,9].

Sonocatalysts, primarily composed of metals or metal oxides, are widely utilized in environmental remediation and therapeutic applications to facilitate chemical reactions and degrade persistent contaminants. Among these materials, titanium dioxide (TiO_2_) is one of the most extensively studied in sonocatalysis [6,7,10,11]. Recent research has aimed to enhance the efficiency of TiO_2_-based sonocatalytic processes and expand their applications in both medical and environmental fields [12,13,14,15,16,17,18]. Incorporating TiO_2_ nanoparticles (NPs) with noble metals, such as gold (Au), has been shown to improve their sonocatalytic performance [19,20,21]. Noble metals not only extend the absorption spectrum through surface plasmon resonance but also serve as electron sinks, facilitating electron transfer and reducing electron–hole recombination.

Gold nanoclusters (Au NCs), tiny aggregates of Au atoms with diameters smaller than 2 nm, exhibit unique properties distinct from bulk gold and larger plasmonic Au NPs [22,23,24]. These clusters display unique electronic states due to quantum-size effects. Structurally, Au NCs consist of a gold atom core surrounded by a protective layer of organic ligands. Their electronic structures, characterized by specific energy levels such as the highest occupied molecular orbital (HOMO) and lowest unoccupied molecular orbital (LUMO), vary with cluster size and significantly influence their physicochemical properties. As innovative nanomaterials, Au NCs hold great potential in catalysis and luminescence, primarily due to their size-dependent properties and exceptional stability [25,26,27].

In our previous research, we explored the sonocatalytic potential of Au NCs [28,29]. Specifically, we synthesized Au_144_ NC/TiO_2_ composites by integrating Au_144_ NCs with TiO_2_ NPs. The composites exhibited significantly enhanced sonocatalytic activity compared to TiO_2_ alone under ultrasonication at 1 MHz [30]. The sonocatalytic activation of the Au_144_/TiO_2_ composite is primarily driven by the generation of high-energy cavitation bubbles during ultrasonication. The characteristics of these cavitation bubbles, which are influenced by the power and frequency of ultrasonication, play a crucial role in determining the sonocatalytic performance of the composite. Despite the promising results, the current literature provides limited insight into how ultrasonication frequency and power affect the sonocatalytic efficiency of Au NC/TiO_2_-based catalysts. To address this gap, our ongoing research aims to optimize the sonocatalytic performance of Au_144_/TiO_2_ composites by systematically refining ultrasonic irradiation parameters, including frequency, intensity, and catalyst concentration. By identifying the optimal conditions for ultrasonic catalysis, we aim to maximize the efficiency of the composites. Additionally, we investigated the size-dependent effects of Au NCs (including plasmonic Au NPs) on the performance of Au NC/TiO_2_-based catalysts. Specifically, we compared Au_144_ NCs, Au_25_ NCs, and plasmonic Au NPs. These findings have the potential to broaden the application of these advanced sonocatalytic materials in diverse industrial and environmental contexts.

## 2. Results and Discussion

### 2.1. Preparation of Au_144_/TiO_2_ Nanocomposite

**Figure 1:**
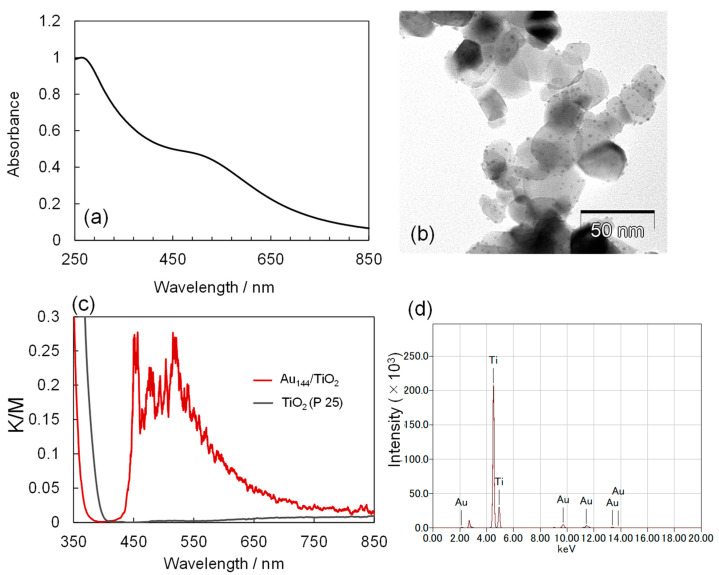
(**a**) UV–Vis absorption spectrum of Au_144_ NCs. (**b**) TEM image of Au_144_ (3 wt.%)/TiO_2_. (**c**) Absorption spectrum of Au_144_ (3 wt.%)/TiO_2_. (**d**) XRF spectrum of Au_144_ NC (3 wt.%)/TiO_2_.

The Au_144_/TiO_2_ nanocomposite was prepared by adsorbing Au_144_ NCs onto TiO_2_ NPs. The UV–Vis absorption spectrum of the Au_144_(pMBA)_60_ NCs exhibited a peak at 280 nm, corresponding to 4–mercaptobenzoic acid (pMBA), along with a broad peak at 520 nm, as shown in Figure 1a. These spectral features are consistent with previously reported characteristics of Au_144_(pMBA)_60_ [30,31]. TEM analysis confirmed that the Au_144_ NCs, comprising 3 wt.%, were successfully loaded onto the TiO_2_ NPs without signs of aggregation. The observed particle size was less than 2 nm (Figure 1b). The reflectance spectra of Au_144_ NCs (3.0 wt.%)/TiO_2_ were evaluated using the Kubelka–Munk (K–M) function, as shown in Figure 1c. This analysis demonstrated UV–vis absorption attributed to the loaded Au_144_ NCs, which was notably absent in pristine TiO_2_. Additionally, X-ray fluorescence (XRF) measurements conducted on the Au_144_ NC (3.0 wt.%)/TiO_2_ powder samples determined the gold content to be 2.6 ± 0.2 wt.% (Figure 1d).

### 2.2. Sonocatalysis of TiO_2_ vs. Au_144_/TiO_2_

**Figure 2:**
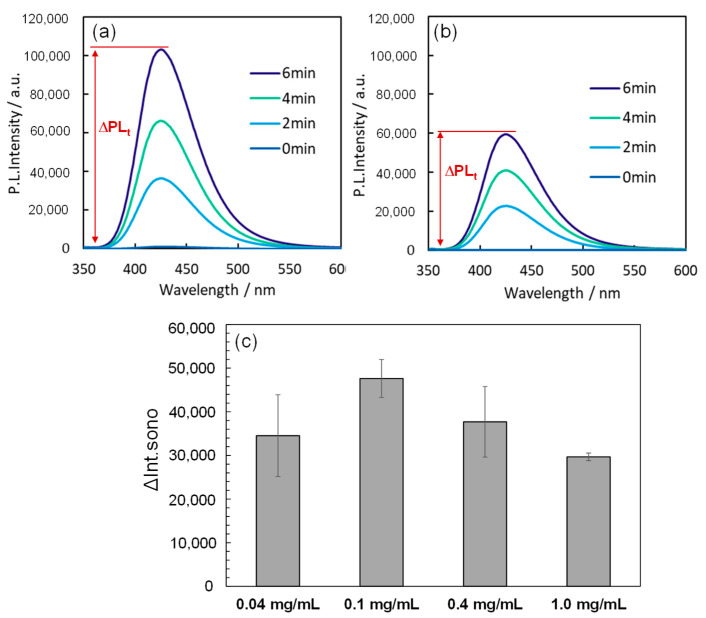
Fluorescence spectra of a NaTA solution during ultrasonic irradiation for 6 min at 430 kHz and 5 W, with (**a**) Au_144_ NC (3 wt.%)/TiO_2_ and (**b**) TiO_2_ alone. Catalyst concentration: 0.1 mg/mL. (**c**) Fluorescence intensity increase (ΔInt_sono_) at 425 nm after 6 min of ultrasonic irradiation with varying concentrations of Au_144_ NC (3 wt.%)/TiO_2_: 0.04 mg/mL, 0.1 mg/mL, 0.4 mg/mL, and 1.0 mg/mL.

When ultrasound was applied to a reaction solution containing the Au_144_/TiO_2_ sonocatalyst, two primary pathways for the generation of •OH radicals were identified: (i) thermal cleavage of water at localized hot spots, and (ii) water oxidation facilitated by the ultrasonically excited sonocatalysts [6,7,8,9]. To evaluate the sonocatalyst’s ability to produce •OH radicals, we measured the fluorescence intensity increase at 425 nm in a disodium terephthalate (NaTA) solution under ultrasonic conditions at 430 kHz and 5 W, both with and without the sonocatalyst. The presence of •OH radicals was detected using NaTA, which reacts with •OH radicals to form fluorescent 2-hydroxy disodium terephthalate (HTA) [30,32,33]. The observed increase in fluorescence intensity in the catalyst-containing reaction solution (ΔPL_t_) was compared to that in the absence of the catalyst (ΔPL_0_). The difference, ΔInt_sono_ = ΔPL_t_ − ΔPL_0_, represents the net contribution of the sonocatalyst to •OH radical generation. This parameter, ΔInt_sono_, is depicted in Figure 2a,b and serves as a quantitative indicator of sonocatalytic activity. The fluorescence intensity increase in the presence of Au_144_/TiO_2_ was higher than that observed for TiO_2_ alone. Our previous study demonstrated that Au_144_ NCs alone do not contribute to an increase in •OH radical production under sonication conditions [30]. These findings demonstrate that the deposition of Au_144_ NCs onto TiO_2_ NPs enhances the sonocatalytic activity, particularly in the generation of •OH radicals.

Highly energetic ultrasonic cavitation can potentially damage the Au_144_/TiO_2_ catalyst. However, our previous study has demonstrated that ultrasound treatment does not compromise the structural integrity of the catalyst, as confirmed by the diffuse reflectance spectra of the Au_144_/TiO_2_ catalyst before and after ultrasonic irradiation [30]. Furthermore, TEM images of the Au_144_ (3 wt.%)/TiO_2_ catalyst were acquired following ultrasonic irradiation for 6 min at 430 kHz and 5 W. While slight particle growth was observed (indicated by arrows in Figure S1), no significant aggregation of Au NCs was detected. The stability of the Au_144_/TiO_2_ catalyst during ultrasonic irradiation is attributed to the coordination of pMBA ligands on the TiO_2_ surface. Specifically, the pMBA ligands attach to TiO_2_ via their carboxyl groups, which deprotonate and bond to the TiO_2_ surface in a bidentate coordination mode [34,35]. This interaction likely plays a critical role in preserving the stability of the Au_144_/TiO_2_ catalyst under the current ultrasonic conditions.

Understanding the dependence of sonocatalytic activity on catalyst concentration is critical for optimizing reaction conditions. An appropriate catalyst concentration ensures maximum •OH radical generation while avoiding potential drawbacks such as light scattering or shielding effects that can occur at higher concentrations. To identify the optimal concentration for the Au_144_/TiO_2_ catalyst, we evaluated its performance at varying concentrations. We measured ΔPL_t_ over a 6 min period of ultrasonic irradiation at a frequency of 430 kHz and an intensity of 5.0 W on reaction solutions containing different concentrations of Au_144_/TiO_2_ (0.04, 0.1, 0.4, and 1.0 mg/mL). Plots of ΔInt_sono_ over the 6 min duration of irradiation are presented in Figure 2c. Across all reaction solutions, a notable increase in ΔInt_sono_ was observed, indicating the oxidation of NaTA to HTA, accompanied by the generation of •OH radicals via Au_144_/TiO_2_. The ΔInt_sono_ values followed the order 0.1 mg/mL > 0.4 mg/mL > 0.04 mg/mL > 1.0 mg/mL, suggesting that the catalytic activity is maximized at a concentration of 0.1 mg/mL. This behavior may be explained by the mechanism of sonocatalyst photoexcitation via sonoluminescence light absorption [32]. At optimal concentrations, the catalyst efficiently absorbs sonoluminescence light, resulting in enhanced •OH radical generation. However, at excessive concentrations, the catalyst may block the sonoluminescence light, limiting the excitation to catalyst particles near the sonoluminescence source. Consequently, the ability to generate •OH radicals decreases as the concentration increases beyond 0.4 mg/mL. Based on these findings, the optimal concentration of the Au_144_/TiO_2_ catalyst was determined to be 0.1 mg/mL, and this concentration was maintained for all subsequent experiments to ensure maximum sonocatalytic activity.

### 2.3. Ultrasound Frequency and Power Dependence

The efficiency of sonocatalytic reactions is highly dependent on ultrasonic frequency and power, as these parameters directly influence the generation and collapse of cavitation bubbles. The cavitation dynamics vary with frequency and power, affecting the energy distribution and localized conditions at cavitation hot spots [6,7,8,9]. Understanding the optimal frequency and power for the Au_144_/TiO_2_ sonocatalyst is crucial for maximizing •OH radical production and ensuring efficient sonocatalytic performance. Furthermore, investigating these parameters provides insight into the mechanisms of ultrasonic activation and the role of cavitation in sonocatalysis.

**Figure 3:**
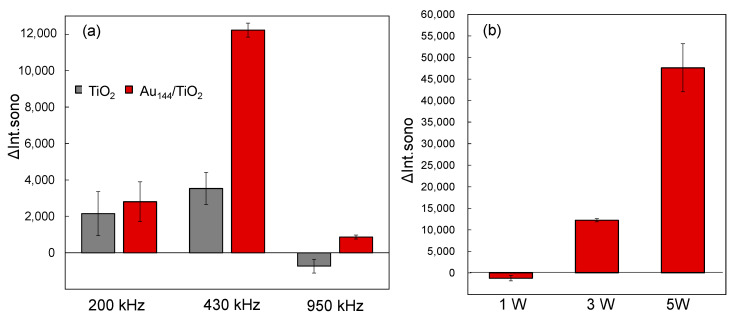
(**a**) Fluorescence intensity increase (ΔInt_sono_) at 425 nm after ultrasonic irradiation at 3 W for 6 min with TiO_2_ alone and Au_144_ (3 wt.%)/TiO_2_ at different ultrasonic frequencies: 200 kHz, 430 kHz, and 950 KHz. Catalyst concentration: 0.1 mg/mL. (**b**) Fluorescence intensity increase (ΔInt_sono_) at 425 nm after ultrasonic irradiation at 430 kHz for 6 min with Au_144_ (3 wt.%)/TiO_2_ at varying ultrasonic power levels: 1 W, 3 W, and 5 W. Catalyst concentration: 0.1 mg/mL.

We investigated the influence of ultrasonic frequencies on ΔInt_sono_ at 200 kHz, 430 kHz, and 950 kHz, as shown in Figure 3a. For all reaction solutions containing Au_144_/TiO_2_, the ΔInt_sono_ values were consistently higher than those of pristine TiO_2_. The observed effect of ultrasonic frequency on enhanced sonocatalytic activity followed the order 430 kHz > 200 kHz > 950 kHz. This trend indicates that the generation rate of •OH radicals was maximized at 430 kHz, driven by the superior sonocatalytic performance of the Au_144_/TiO_2_ catalyst. The frequency dependence can be attributed to variations in cavitation and sonoluminescence efficiency at different frequencies.

To further evaluate the impact of ultrasonic power on sonocatalytic performance, we analyzed ΔInt_sono_ at 430 kHz with power levels of 1.0 W, 3.0 W, and 5.0 W, as shown in Figure 3b. The power levels represent electrical power inputs. The results revealed a power-dependent increase in ΔInt_sono_, following the order 5.0 W > 3.0 W > 1.0 W. However, power levels exceeding 5 W did not significantly enhance ΔInt_sono_ and posed risks of equipment damage, such as deterioration of the ultrasonic transmission gel. These observations suggest that 5.0 W represents an optimal power level, balancing effective •OH radical generation with equipment safety. At lower power levels, insufficient cavitation activity limits radical production, while excessively high power levels may lead to inefficiencies caused by energy dissipation or hardware constraints.

### 2.4. Mechanistic Insights into Enhanced Sonocatalytic Activity in Au_144_/TiO_2_

**Figure 4:**
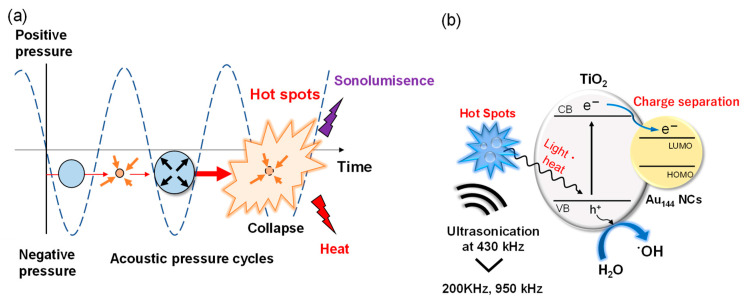
Conceptual diagram illustrating enhanced •OH radical generation in Au_144_/TiO_2_ sonocatalyst system. (**a**) Thermal and sonoluminescence generation at cavitation hot spots during ultrasonication. (**b**) Enhanced mechanism for •OH radical generation involving the migration of excited electrons from TiO_2_ to Au_144_ NCs, simultaneously inhibiting electron–hole recombination.

The enhanced sonocatalytic activity of Au_144_/TiO_2_ compared to TiO_2_ alone demonstrates the enhanced effects of Au_144_ NCs deposited on TiO_2_. Figure 4 illustrates the proposed mechanisms driving enhanced •OH radical generation in the Au_144_/TiO_2_ sonocatalyst system under ultrasonic irradiation. This model highlights two key processes, ultrasonic cavitation-induced excitation and charge separation, which together contribute to the system’s superior sonocatalytic performance. During ultrasonic irradiation, acoustic pressure cycles lead to the formation, growth, and collapse of cavitation bubbles, as depicted in Figure 4a. The collapse of these bubbles creates extreme localized conditions, such as transiently high temperatures and pressures at cavitation hot spots. These conditions induce thermal effects and UV sonoluminescence in the wavelength range of 200–400 nm [36], which promote the excitation of electrons in TiO_2_ from the valence band (VB) to the conduction band (CB).

Hydroxyl radicals (•OH) are generated through the thermal decomposition of water under the extremely high temperatures and pressures created during the adiabatic collapse of ultrasonic cavitation bubbles. This process forms localized hot spots characterized by transiently elevated temperatures and pressures, resulting in the homolytic cleavage of water molecules and subsequent formation of •OH radicals [37].

**Figure 5:**
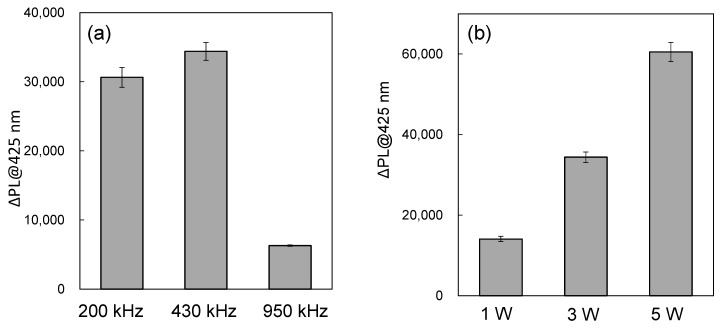
(**a**) Fluorescence intensity increase (ΔPL) in NaTA aqueous solutions (no catalyst) at 425 nm after 6 min of ultrasonic irradiation at 3 W, varying the ultrasonic frequencies of 200 kH, 430 kHz, and 950 kHz. (**b**) Fluorescence intensity increase (ΔPL) in NaTA aqueous solutions (no catalyst) at 425 nm after 6 min of ultrasonic irradiation at 450 kHz, varying the ultrasonic power of 1 W, 3 W, and 5 W.

In this study, we monitored the generation of •OH radicals in water (without the use of catalysts) under ultrasonic irradiation at varying frequencies (200 kHz, 430 kHz, and 950 kHz) and power levels (1 W, 3 W, and 5 W) using the NaTA method, as shown in Figure 5. The increased absorbance intensity observed at 430 kHz suggests a higher occurrence of cavitation events, more intense bubble collapses, or both, which collectively enhance hydroxyl radical production at this frequency. Consequently, the higher generation of •OH radicals at 430 kHz reflects more favorable cavitation conditions, such as an increased number of bubbles or more violent bubble collapses, conducive to the efficient excitation of the Au_144_/TiO_2_ system. This enhanced excitation likely contributes to the maximum sonocatalytic activity observed at 430 kHz.

Previous studies have shown that sonoluminescence intensity peaks at intermediate frequencies, such as approximately 360 kHz, while it is diminished at both higher frequencies (e.g., around 1000 kHz) and lower frequencies (e.g., around 100 kHz) [38,39,40]. These results suggest that the efficient ultrasonic excitation of the Au_144_/TiO_2_ catalyst observed at 430 kHz is likely driven by cavitation-induced sonoluminescence.

The above results highlight the critical role of cavitation dynamics in activating the Au_144_/TiO_2_ catalyst and enhancing its sonocatalytic efficiency. Future studies will be needed on directly examining bubble dynamics using advanced techniques, such as high-speed imaging or sonoluminescence measurements, to establish a clearer link between bubble behavior and sonocatalytic activity across different frequencies [41]. These investigations are expected to provide deeper mechanistic insights into the optimal performance observed at 430 kHz.

As for the deposition of Au_144_ NCs on TiO_2_, the excited electrons in TiO_2_ are subsequently transferred to the Au_144_ NCs. This efficient electron migration suppresses electron–hole recombination, thereby prolonging the lifetime of charge carriers. The enhanced charge separation facilitates more effective water oxidation, increasing the production of •OH radicals, as depicted in Figure 4b.

**Figure 6:**
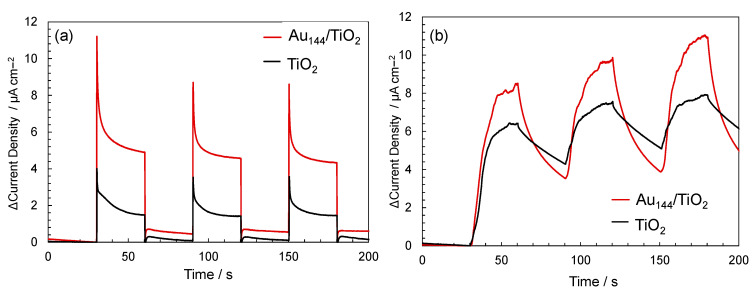
(**a**) Transient photocurrent profile and (**b**) transient sonocurrent profile comparison between TiO_2_ and Au_144_ (3 wt%)/TiO_2_.

To validate this hypothesis, transient photocurrent and sonocurrent measurements were conducted for Au_144_/TiO_2_ composites and bare TiO_2_. Transient photocurrent and sonocurrent measurements serve as direct experimental evidence for enhanced charge separation in photocatalytic and sonocatalytic systems. A higher photocurrent or sonocurrent indicates more efficient charge carrier generation and reduced recombination of electron–hole pairs. These measurements are thus widely used to evaluate the ability of a material to facilitate charge separation under irradiation or ultrasonic excitation [42]. As shown in Figure 6a, Au_144_/TiO_2_ exhibited a higher photocurrent density under light exposure compared to bare TiO_2_, indicating improved charge separation and transfer. Similarly, transient sonocurrent measurements at 430 kHz (Figure 6b) demonstrated enhanced electron generation in Au_144_/TiO_2_ compared to TiO_2_ alone. These findings provide strong evidence that Au_144_ NCs play a crucial role in facilitating charge separation and improving the overall sonocatalytic performance of the system.

### 2.5. Size Dependence in Sonocatalytic Activity in Au/TiO_2_

The size of AuNCs deposited on semiconductors, such as TiO_2_, is a critical factor influencing the catalytic performance of composite materials. Optimizing the size of AuNCs can significantly improve light absorption, charge separation, active site availability, and overall catalytic efficiency [27,43]. The transition from metallic to molecular behavior in Au nanoparticles significantly influences their catalytic properties. Previous studies reveal that this transition occurs between Au_333_ and Au_144_, corresponding to sizes of approximately 1.7–2.3 nm. Larger nanoparticles, such as AuB_520_ and AuB_940_, exhibit metallic characteristics, whereas Au_144_ and smaller particles demonstrate molecular-like behavior. This size-dependent transition affects the catalytic performance of Au_25_, Au_38_, Au_144_ (1.7 nm), and Au_333_ (2.3 nm), as observed in both CO oxidation and the electrocatalytic oxidation of alcohol. Among these, Au_144_ displayed the highest catalytic performance, emphasizing the critical role of nanoparticle size and electronic structure in optimizing catalytic efficiency [44].

**Figure 7:**
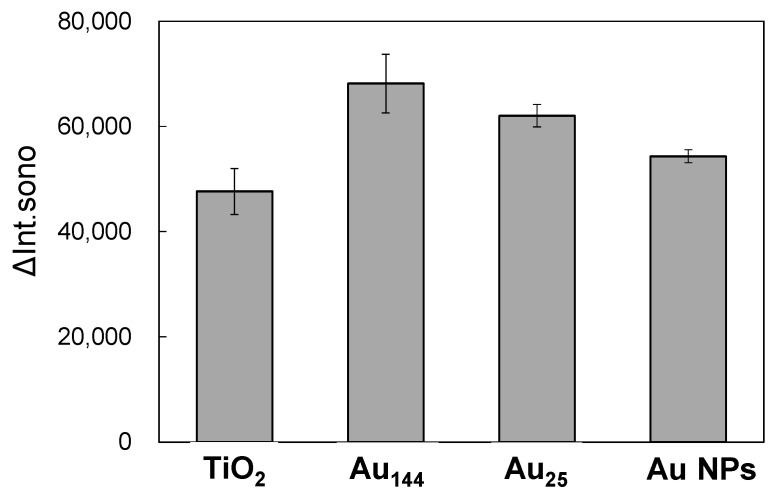
Fluorescence intensity increase (ΔInt_sono_) at 425 nm after ultrasonic irradiation at 5 W for 6 min with TiO_2_ alone and Au (3 wt.%)/TiO_2_ at an ultrasonic frequency of 430 kHz. Catalyst concentration: 0.1 mg/mL.

This study examines how the size of Au NCs influences the sonocatalytic performance of Au/TiO_2_ catalysts. The catalytic efficiency of variously sized Au NCs, including plasmonic Au nanoparticles (NPs), was evaluated (Figure 7). The ΔInt_sono_ values, which represent sonocatalytic efficiency, followed the order Au_144_ > Au_25_ > plasmonic Au NPs > bare TiO_2_. These findings highlight the superior performance of Au NCs compared to plasmonic Au NPs within the Au/TiO_2_ catalyst system. Ultra-small Au NCs (<2 nm) possess exceptionally high surface energy at the atomic level, significantly enhancing their catalytic activity. In contrast, larger Au NPs (~2.4 nm), with a relatively reduced reactive surface area, demonstrate diminished catalytic performance.

Among the studied catalysts, Au_144_ NCs exhibited the highest performance, surpassing Au_25_ NCs by achieving an optimal balance between electron migration and charge separation. Smaller clusters, such as Au_25_ NCs, are known to inject photoexcited electrons into the TiO_2_ conduction band upon light absorption [45]. To test this hypothesis, we conducted additional experiments to evaluate photocatalytic •OH radical generation under visible light irradiation, selectively exciting Au_144_ NCs without activating TiO_2_. The results revealed no detectable •OH radical generation in the Au_144_ NC/TiO_2_ system under visible light (Figure S2), whereas the Au_25_ NCs/TiO_2_ system did exhibit •OH radical production (Figure S3). This finding suggests that excited electrons from Au_144_ NCs make a negligible contribution to •OH radical generation in this system. This behavior is likely due to the efficient electron injection capability of Au_25_ NCs into TiO_2_. While smaller clusters like Au_25_ can efficiently inject electrons, they are prone to increased charge recombination due to bidirectional electron transfer, which can diminish the overall efficiency of photocatalytic and sonocatalytic reactions. In contrast, larger clusters like Au_144_ may strike a better balance between electron injection and charge recombination, resulting in enhanced overall performance for specific applications.

The transition from Au NCs to plasmonic Au NPs, as well as the size-dependent effects and electronic structure variations, remains only partially understood in the Au/TiO_2_ sonocatalyst system. Future research should focus on elucidating the interplay between size and electronic structure using advanced spectroscopy and computational modeling. These insights will deepen our understanding of Au-based sonocatalytic systems, emphasizing the critical role of Au NC size and electronic structure in enhancing sonocatalytic performance.

## 3. Materials and Methods

### 3.1. Reagents

Hydrogen tetrachloroaurate (III) tetrahydrate (HAuCl_4_·4H_2_O) and methanol were sourced from FUJIFILM Wako Pure Chemical Corporation (Osaka, Japan). Disodium terephthalate (NaTA) and 4–mercaptobenzoic acid (pMBA) were purchased from Tokyo Chemical Industry Co., Ltd. (Tokyo, Japan). Sodium borohydride (99%) (NaBH_4_) was procured from Sigma-Aldrich (St. Louis, MO, USA). The TiO_2_ utilized was Aeroxide^®^P25. Deionized water was prepared using a water distillation apparatus (Aquarius RFD250; ADVANTEC, Tokyo, Japan).

### 3.2. Instruments

UV–visible (UV–vis) absorption spectroscopy and steady-state fluorescence spectroscopy were performed using a JASCO V-670 spectrophotometer (JASCO Corp., Tokyo, Japan) and RF-6000 spectrofluorometer (Shimadzu Corp., Kyoto, Japan), respectively. Diffuse reflectance UV–vis spectra were acquired using an Ocean Optics DH-2000-BAL deuterium–tungsten–halogen light source and an Ocean Optics USB4000 compact fiber optic spectrometer (Ocean Optics, Dunedin, FL, USA). The Au loading on TiO_2_ NPs was quantified using an energy-dispersive X-ray fluorescence (XRF) spectrometer (JSX-1000S Element Eye; JEOL Ltd., Tokyo, Japan). The crystal structure of the TiO_2_ NP powder was analyzed by X-ray diffractometry (XRD, D2 Phaser, Bruker AXS GmbH, Karlsruhe, Germany), employing a Cu-Kα radiation source (λ = 1.5406 Å) over a 2θ range of 10–80°, at an accelerating voltage of 30 kV and a current of 10 mA. The size and morphology of Au_144_/TiO_2_ were characterized using transmission electron microscopy (TEM; JEOL JEM2100 microscope operating at 200 kV, JEOL Ltd., Tokyo, Japan).

### 3.3. Synthesis of Au_144_(pMBA)_60_

According to our previous study [30], Au_144_(pMBA)_60_ NCs (Au_144_ NCs) were synthesized at an ambient temperature by mixing HAuCl_4_ aqueous solution (25 mM, 3 mL), pMBA solution (75 mM, 3 mL), deionized water (6.5 mL), and methanol (12.5 mL), with methanol comprising 50% of the total volume of 25 mL. After stirring the solution overnight until it turned colorless, NaBH_4_ was added in cold deionized water (500 mM, 227 µL) and the mixture was stirred for an additional 2 h. After the addition of methanol, the NCs were isolated by centrifugation and the purification step was repeated. Finally, the NCs were dried under reduced pressure to obtain the purified product.

### 3.4. Synthesis of Au_25_(pMBA)_18_

The synthesis of Au_25_(pMBA)_18_ nanoclusters (Au_25_ NCs) was conducted at room temperature in air, following a previously reported method [30]. The successful synthesis of Au_25_(pMBA)_18_ was confirmed through UV–vis spectroscopy, which revealed two prominent absorption bands at 450 nm and 670 nm, along with a broad shoulder around 800 nm. These spectral features align well with previously reported characteristics of Au_25_ NCs [30].

### 3.5. Synthesis of Au Nanoparticles

Gold nanoparticles (Au NPs) with a size of 2.4 nm were synthesized under ambient conditions and at room temperature following the reported procedure [46]. Initially, 2.7 mL of the 28 mM HAuCl_4_ solution and 2.4 mL of the 95 mM pMBA solution were mixed. This was followed by the gradual addition of 20 mL of the 106 mM NaOH solution. The resulting reaction mixture was stirred continuously for 20 h to ensure complete interaction of the components. The final concentrations of the key reactants in the reaction mixture were as follows: HAuCl_4_, 3 mM; pMBA, 9 mM; and NaOH, 100 mM. To reduce the gold ions, 28.3 mg of NaBH_4_ was dissolved in 5 mL of cold deionized water, yielding a 150 mM NaBH_4_ solution. Subsequently, a 0.25 mL aliquot of this NaBH_4_ solution was added to the reaction mixture, maintaining a molar ratio of NaBH_4_:Au = 1:2. The reaction was stirred overnight to facilitate reduction. To complete the synthesis, 29.2 mg of NaCl was added to the reaction mixture to achieve a final NaCl concentration of 10 mM. Methanol (80% *v*/*v*) was then introduced and the mixture was centrifuged at 6000 rpm for 10 min. The resulting precipitate was collected, dried, and stored, yielding the final Au NPs. The successful synthesis of Au NPs was confirmed through UV–vis spectroscopy, which revealed a plasmonic absorption band at around 520 nm [37].

### 3.6. Preparation of Au/TiO_2_ Composites

Following the method described in our previous report [30], a Au_144_ NC-supported TiO_2_ composite was fabricated at ambient temperature. In a reaction tube, 8 mL of an aqueous TiO_2_ dispersion (1 mg/mL) was combined with 80 μL of an aqueous Au_144_ NC solution (3.0 mg/mL). The mixture was stirred at 800 rpm for 17 h to promote the adsorption of Au_144_ NCs onto the TiO_2_ particles, yielding a composite with a 3 wt.% loading of Au_144_ NCs on TiO_2_. After stirring, the mixture was centrifuged at 6000 rpm for 15 min to separate the supernatant, leaving the Au_144_ NC (3 wt.%)/TiO_2_ composite as a precipitate. Similarly, composites of Au_25_ NCs (3 wt.%) and Au NPs (3 wt.%) supported on TiO_2_ were prepared using the same procedure.

### 3.7. Evaluation of Sonocatalytic Activity

The sonocatalytic activity of Au/TiO_2_ nanocomposites was assessed by measuring the generation of •OH radicals from water under ultrasonic stimulation. The presence of •OH radicals was detected using disodium terephthalate (NaTA), which reacts with •OH radicals to form fluorescent 2-hydroxy disodium terephthalate (HTA) [30]. The production of •OH radicals was monitored by measuring the fluorescence intensity of HTA at 425 nm (excitation at 315 nm). To ensure consistent ultrasonic transmission and eliminate air gaps, an ultrasonic transmission gel was applied between the transducer and a plastic dish during the experiment. The experimental setup was maintained at a constant temperature of 18 ± 2 °C using a temperature-controlled water bath equipped with a precision thermostat. The reaction solution was subjected to ultrasonic irradiation at intervals of 0, 2, 4, and 6 min using a QUAVA Mini QR-003 ultrasonic device (Kaijo, Hamura, Japan) at frequencies of 200 kHz, 430 kHz, and 950 kHz. After each irradiation, the mixture was centrifuged at 14,000 rpm for 10 min to separate the catalyst. The fluorescence intensity of the supernatant was measured at 425 nm to quantify the production of •OH radicals.

### 3.8. Transient Photocurrent/Sonocurrent Measuremetns in TiO_2_ and Au_144_ (3 wt%)/TiO_2_

To determine whether supporting Au_144_ NCs on TiO_2_ improves charge separation, transient photocurrent and ultrasonic current responses were measured using a three-electrode cell connected to a potentiostat (BAS, ALS611 DE). The measurement parameters were as follows: mode—amperometry i-t curve; initial potential—−0.1 V; sample interval—0.1 s. A Pt mesh served as the counter electrode and an Ag/AgCl electrode (BAS, RE-1B) was used as the reference electrode. The working electrode was prepared by dispersing 5 mg of the catalyst in a mixture of 375 µL of water containing 20 wt.% Nafion (50 µL) and 125 µL of 2-propanol. A 40 µL aliquot of this dispersion was applied onto a 1 cm^2^ fluorine-doped tin oxide (FTO) glass conductive surface and dried under reduced pressure overnight.

All measurements were performed in a 0.2 M Na_2_SO_4_ aqueous solution. The transient photocurrent response was recorded under UV light (394 nm, 22.46 mW/cm^2^) using a UV Spotlight Source (L5662, Hamamatsu Photonics, Shizuoka, Japan). For the transient ultrasonic current response, a 200 kHz, 50 W ultrasound device (QUAVA mini QR-003, Kaijo Corporation, Hamura, Japan) was employed. To minimize temperature elevation in the electrolyte due to ultrasonic irradiation, the reaction vessel was placed on a cooling plate submerged in a water-filled container.

## 4. Conclusions

In this study, we investigated the sonocatalytic capabilities of Au_144_/TiO_2_ composites, focusing on the generation of •OH radicals under varying ultrasonic frequencies, power levels, and NC sizes. Our findings demonstrate that the Au_144_/TiO_2_ composite exhibits superior sonocatalytic performance, particularly at an ultrasonic frequency of 430 kHz and a power setting of 5.0 W. This heightened activity is attributed to the optimal cavitation dynamics achieved at 430 kHz, which maximizes localized thermal effects and sonoluminescence, facilitating efficient •OH radical generation.

The size dependence of the Au NCs was also found to play a critical role in determining the sonocatalytic performance in Au/TiO_2_ composites. The comparative analysis revealed a clear efficiency trend: Au_144_ > Au_25_ > plasmonic Au NPs > bare TiO_2_. Larger clusters like Au_144_ demonstrated superior activity due to their discrete electronic structures, which enable better energy alignment with the TiO_2_ conduction band, facilitating enhanced charge separation and prolonged carrier lifetimes. In contrast, plasmonic Au NPs were less efficient under ultrasonic activation.

This research highlights the practical potential of the Au_144_/TiO_2_ system for applications in environmental remediation and medical treatments. The insights gained into the interplay between ultrasonic frequency, cavitation dynamics, and nanocluster size offer a pathway for developing advanced sonocatalytic materials. Ultimately, this study contributes to the broader field of chemical physics by emphasizing the significance of NP-enhanced sonocatalysis in addressing global challenges, including pollution control and sustainable healthcare solutions.

## Data Availability

Data are contained within the article and Supplementary Materials.

## Supplementary Materials

The following supplementary materials can be downloaded at https://www.mdpi.com/article/10.3390/molecules30030541/s1: Figure S1. TEM image of Au_144_ (3 wt.%)/TiO_2_ catalyst after ultrasonic irradiation for 6 min at 430 kHz and 5 W; Figure S2. Fluorescence spectra of a NaTA solution under xenon lamp (150 W, λ > 420 nm): TiO_2_ alone and Au_144_ NC (3 wt.%)/TiO_2_; Figure S3. Fluorescence spectra of a NaTA solution under xenon lamp (150 W, λ > 420 nm): TiO_2_ alone and Au_25_ NC (3 wt.%)/TiO_2_.
