# Supplementary material for: Ultrasonic Activation of Au Nanoclusters/TiO2: Tuning Hydroxyl Radical Production Through Frequency and Nanocluster Size
Source: Molecules. 2025 Jan 24;30(3):541. doi: 10.3390/molecules30030541 (PMC11819690; doi:10.3390/molecules30030541)
Supplement: Supplementary file 1 [file molecules-30-00541-s001.zip › molecules-3388643-supplementary.pdf]

Supplementary Materials

Article

Ultrasonic Activation of Au nanoclusters/TiO_2: Tuning Hydroxyl Radical Production through Frequency and Nanocluster Size

Takaaki Tsurunishi, Yuzuki Furui, and Hideya Kawasaki*

Department of Chemistry and Materials Engineering, Kansai University, 3-3-35, Yamate-cho, Suita,

Osaka 564-8680, Japan

Correspondence: hkawa@kansai-u.ac.jp(H.K.)

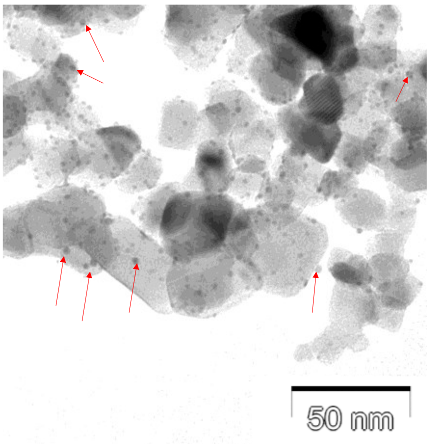

Fig. S1 TEM image of Au_{144} (3 wt.%)/TiO_2 catalyst after ultrasonic irradiation for 6 minutes at 430 kHz and 5 W. While slight particle growth was observed (indicated by arrows), no significant aggregation of Au NCs was detected.

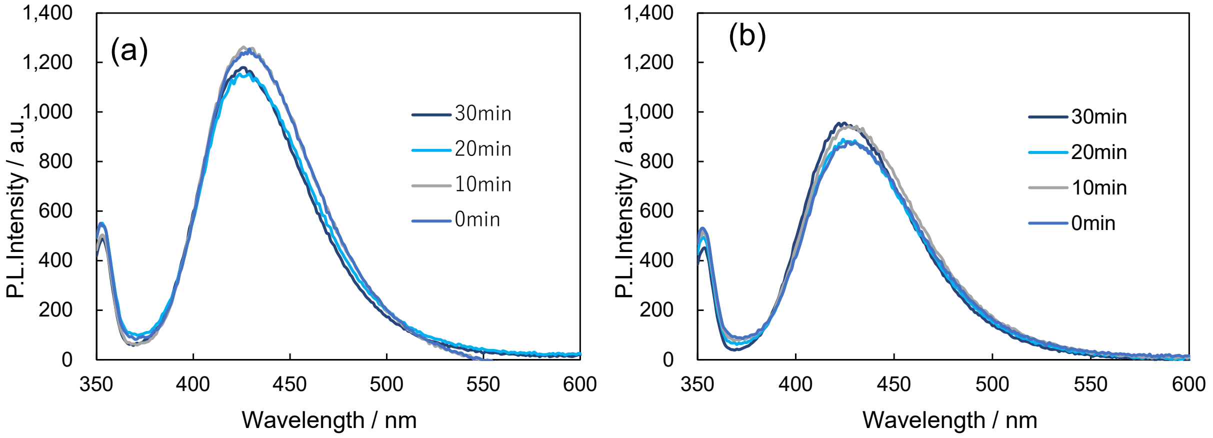

Fig. S2 Fluorescence spectra of a NaTA solution during xenon lamp (150W, λ>420 nm): (a) TiO_2 alone and (b)Au_{144} (3 wt.%)/TiO_2.

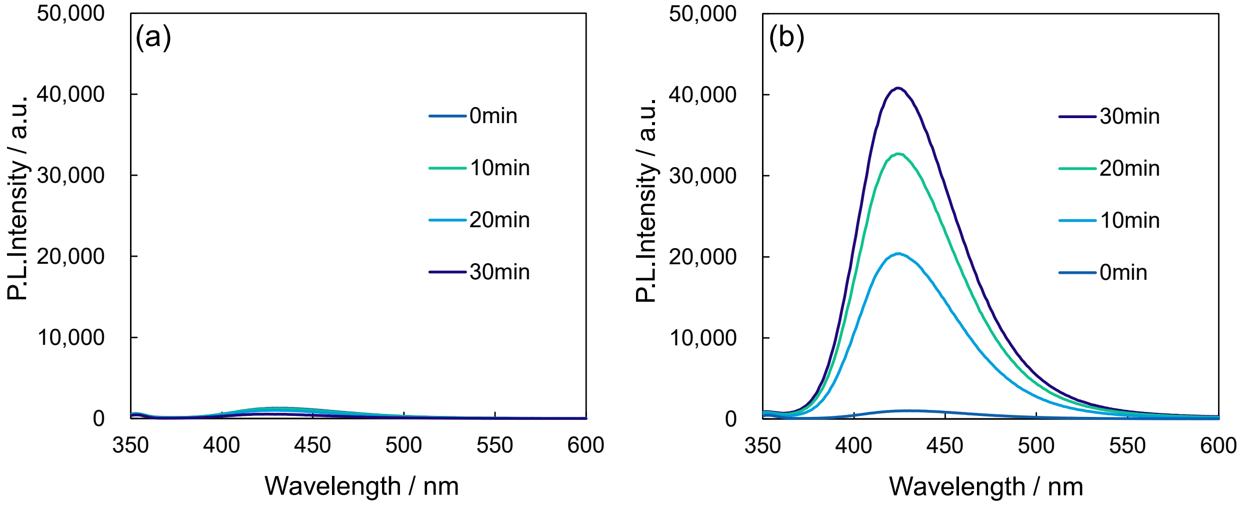

Fig. S3 Fluorescence spectra of a NaTA solution during xenon lamp (150W, λ>420 nm): (a) TiO_2 alone and (b)Au_{25} (3 wt.%)/TiO_2.
